# Trophic ecology of yellowtail rockfish (*Sebastes flavidus*) during a marine heat wave off central California, USA

**DOI:** 10.1371/journal.pone.0251499

**Published:** 2021-05-13

**Authors:** Jennifer A. Chiu, Joseph J. Bizzarro, Richard M. Starr

**Affiliations:** 1 Moss Landing Marine Laboratories, Moss Landing, California, United States of America; 2 Cooperative Institute for Marine, Earth, and Atmospheric Ecosystems, University of California Santa Cruz, Santa Cruz, California, United States of America; 3 Fisheries Ecology Division, Southwest Fisheries Science Center, National Marine Fisheries Service, NOAA, Santa Cruz, California, United States of America; Universidad de Antioquia, COLOMBIA

## Abstract

The yellowtail rockfish, *Sebastes flavidus*, is a widespread and abundant mesopredator in the California Current Large Marine Ecosystem. We utilized stomach content and stable isotope analyses to investigate the trophic ecology of this species at three sites off central California just before (August–October 2013) and during (August and October 2014) a marine heat wave. *Sebastes flavidus* largely consumed pelagic prey (zooplankton and micronekton). Diets were dominated by tunicates (salps and pyrosomes), pelagic crustaceans (euphausiids, hyperid amphipods, larval decapods), and fishes, with the relative contribution of these prey taxa varying spatially (sample location, longitude, depth) and temporally (year, month), based on complementary multivariate analyses. Prey-specific indices demonstrated that individual *S*. *flavidus* diet composition typically was dominated by one of these prey groups, and that prey switching occurred based on the relative availability of prey and their energetic importance. Stable isotope analysis of δ^15^N indicated that the *S*. *flavidus* populations sampled in 2014 had been feeding at an elevated trophic position and more variable prey spectrum relative to 2013, probably as a consequence of greater piscivory and the incorporation of temporal changes in diet composition. Because its opportunistic feeding behavior reflects the dynamism and heterogeneity of the pelagic forage preyscape, *S*. *flavidus* may be an important ecosystem indicator species. For example, the novel incorporation of pyrosomes as a large portion of the diet of *S*. *flavidus* during 2013–2014 directly related to the massive increase in pyrosome abundance in the California Current during the 2014 marine heat wave.

## Introduction

A large, anomalously warm, water mass (known as “the Blob”) appeared off the coast of Alaska, USA in the boreal winter of 2013–2014 and expanded south in subsequent years to Baja California, Mexico [1]. This warm-water anomaly in the northeastern Pacific Ocean persisted for several years and considerably altered pelagic community structure in the California Current ecosystem [2, 3]. In 2013, the California Current experienced strong coastal upwelling, anomalously cold sea surface temperatures (SSTs), and high productivity (i.e., chlorophyll a concentration [4]; however, it transitioned to a warm state (i.e., decreased upwelling, anomalously warm SST, decreased productivity) by August 2014, This condition along the Washington, Oregon, and California coasts, termed a marine heatwave, persisted until August 2016, causing major physical and biological disturbances in the California Current Large Marine Ecosystem [5], and widespread economic impacts. Mass strandings of seabirds, failure of salmonid year-classes, shifts in distributions of fishes and in invertebrate species composition and relative abundance, and increases in nearshore whale entanglements were all reported [1, 6, 7].

The yellowtail rockfish (*Sebastes flavidus*) is a mid-water species that is abundant from central California to Alaska [8]. It has historically been one of the primary species taken in nearshore recreational fisheries off California and is an important commercial target off Oregon and Washington [9]. *S*. *flavidus* typically inhabits coastal waters between depths of 90–180 m, but has been found from surface and intertidal waters to a depth of 549 m. Maximum length, weight, and age are 66 cm (total length), 4.2 kg, and 64 yr, respectively [8].

Because of its widespread occurrence and high relative abundance, *S*. *flavidus* is an important predator in nearshore, midwater regions of the California Current Ecosystem. The diet of *S*. *flavidus* typically consists of a diverse range of planktonic and micronektonic prey items [10–12]. Seasonal dietary shifts among pelagic crustaceans (e.g., euphausiids, copepods, larval decapods) and gelatinous zooplankton have been reported among published studies [10, 12, 13]. This type of seasonal variability in diet composition is common for fishes living in eastern boundary currents, which are greatly affected by fluctuations in physical conditions and productivity over multiple temporal scales [14–17].

In 2013 and 2014, we collected *S*. *flavidus* off central California, USA, as part of a study that evaluated the effectiveness of fishery closures at rebuilding fish populations [18]. The timing of that study enabled us to evaluate the differences between diets of *S*. *flavidus* before and during a marine heat wave, using gut content and stable isotope analyses. Specific objectives of our study were to: 1) characterize diet composition, 2) determine and evaluate sources of dietary variability, 3) identify stable isotope signatures in white muscle tissues of sampled fish, and 4) combine stable isotope ratios with the gut content analysis to construct a holistic description the trophic ecology of *S*. *flavidus*.

## Materials and methods

### Data collection

**Fig 1:**
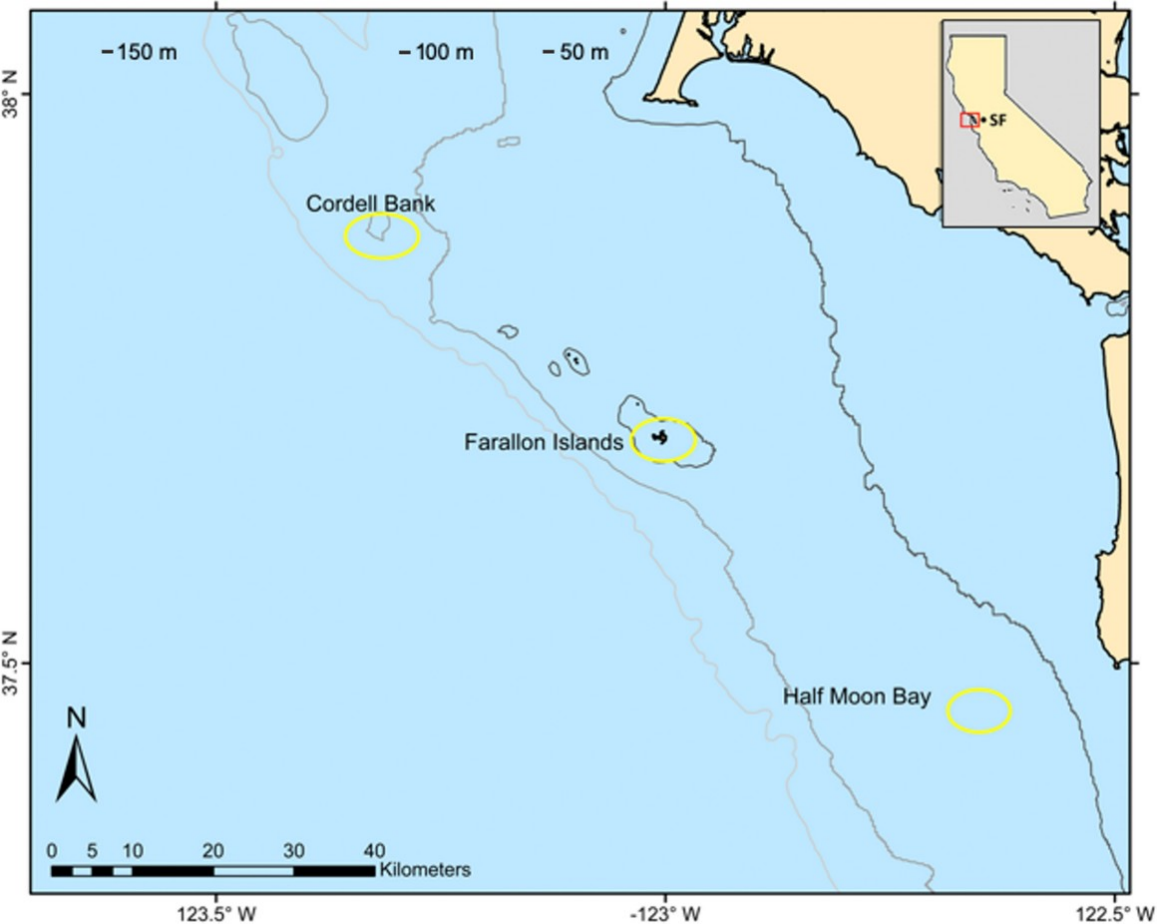
Sampling locations for *Sebastes flavidus* in central California during 2013 and 2014.

*Sebastes flavidus* individuals were obtained from three central California locations: Cordell Bank (COR), Farallon Islands (FAR), and Half Moon Bay (HMB) (Fig 1). The Cordell Bank collection sites were located on the edge of the continental shelf, about 40 km offshore in the center of the California Current and mainly contained high-relief rocky habitats. The Farallon Islands sites also were located on the edge of the continental shelf, approximately 50 km west of San Francisco and in an area influenced by the California Current and by lower salinity water emanating from San Francisco Bay. The Half Moon Bay sites were between 4–20 km offshore and included low relief, rocky habitats in the middle of the continental shelf [18]. Fish were caught at depths ranging from 37–168 m, with a mean depth of 73 m.

We conducted this work under the National Oceanic and Atmospheric Administration (NOAA) Scientific Research Permits SRP-22-2013 and SRP-22-2014. This study was carried out in strict accordance with the San Jose State University (SJSU) Institutional Animal Care and Use Committee (IACUC), which approved the study under protocol #2015-B. All specimens were caught via hook-and-line at each of the three study locations between August and October of 2013 and 2014. Fish with stomach eversions (due to barotrauma) were not retained. Aboard the fishing vessel, each fish was measured to the nearest half centimeter (total length), euthanized using cranial concussion according to Institutional Animal Care and Use Committee protocols, and placed in coolers with ice for transit back to the harbor. Fish stomachs and tissue samples were removed within 24 h of capture, and each individual was sexed. The excised stomachs were placed in Nasco Whirl-Paks. White muscle tissue samples for stable isotope analysis were taken from just below the dorsal fin and placed in cryovials. All samples were labeled with a unique identification number and stored frozen until processing.

### Data analyses

*Sebastes flavidus* stomach samples were thawed and processed in the laboratory, and diet composition was quantified using established dietary metrics. Prey items were identified to the lowest possible taxonomic level, enumerated, and weighed to an accuracy of 0.001 g. When fish samples were well digested, paired parts that were more resistant to digestion (e.g., otoliths, eyes) were grouped by taxon, counted, and halved to estimate a minimum count. Similarly, when crustaceans were well digested, the number of individuals consumed was determined by counting the total number of carapaces [19]. Incidentally ingested materials, such as rocks and parasites were omitted from diet composition estimates. Bait used to catch fish (mantles of cut market squid, *Doryteuthis opalescens*) was easily identifiable in stomachs and also was excluded from analyses. We used the following metrics to evaluate the contributions of food items to fish diets: average percent number (%N, [20]), average percent weight (%W, [20]), prey-specific number (%PN, [21, 22]), prey-specific weight (%PW, [21, 22]), prey-specific index of relative importance (%PSIRI, [22]) and frequency of occurrence (%FO, [20]).

We plotted the number of unique prey categories as a function of the number of stomachs analyzed to determine if enough stomach samples were collected to adequately describe the diet of *S*. *flavidus* in each year and location [23]. Both the lowest possible level of taxonomic identification and higher (generalized) taxonomic distinctions (after [24]) were used to determine sample size sufficiency. Generalized prey categories included: Cephalopoda (Squids), unidentified Crustaceans, Amphipods, Euphausiids, Tunicates (pyrosomes and salps), unidentified Teleosts, Rockfishes (*Sebastes* spp.), and Other organisms, which consisted of rare prey taxa (i.e., gastropods, polychaetes, isopods). Cumulative prey curves were plotted using the software program R (v. 3.3.2) and the Vegan Community Ecology package [25]. We performed a linear regression using the last five points of the curve to test for adequate sample size and determine if the slope (*b*) of the linear regression was ≤ 0.05. If so, the curve was considered to have reached an asymptote [26, 27].

The trophic levels of individual *S*. *flavidus* were determined from the stomach contents following techniques described by [28]:

$$TL_{k}=1+\left(\sum_{j=1}^{n}P_{j}*TL_{j}\right)$$

where *TL*_*k*_ is the trophic level of species *k*, *P*_*j*_ is the proportion of prey category *j* in the diet of species *k*, *n* is the total number of prey categories, and *TL*_*j*_ is the trophic level of prey category *j*. Trophic levels were assigned based on categories described by [29]. We conducted an ANCOVA analysis to determine if there was a significant difference between trophic level and total length by sex.

We used PERMANOVA (with permutation tests of multivariate group dispersions, [30]) to determine which combination of response variables best explained the observed dietary variability of *S*. *flavidus*. Two individual-based abundance estimates, percentage by number (%N) and percentage by weight (%W) were used to generate separate analyses. The Bray-Curtis dissimilarity index was used as the basis for matrix calculations and the PERMANOVA model was permuted 999 times [31]. All multivariate analyses were conducted in R (v. 3.3.2) using the Vegan Community Ecology package [25]. After highly correlated variables (Pearson’s *r* > 0.65, *P* < 0.05) were removed, the explanatory variables used in the PERMANOVA were Location, Year, Month, Longitude, Depth, and Length. All stomach samples containing exclusively Other prey (*n* = 3) were omitted from multivariate analyses. Stomach samples with both Other prey and additional prey taxa (*n* = 5) were recalculated after Other prey contributions were removed. A forward, stepwise approach was used to establish the best overall model, including individual variables and the interaction terms of Location x Year, Year x Month, and Location x Year x Month. For both independent variables and interaction terms, significant factors were ranked by F-statistic values, which indicate the relative magnitude of significant differences among means.

Canonical analysis of principal coordinates (CAP) was performed on the same data sets to supplement the results of the PERMANOVA by determining the relationship between prey categories and response variables. CAP enables the calculation of a constrained ordination using any distance or resemblance measure [32]. A Bray-Curtis dissimilarity matrix was used as the basis for CAP analyses with %N and %W data. Significance of the overall model, each canonical axis, and each response variable was determined by 999 permutations. For each CAP model, a biplot of significant response variables and prey categories along the first two canonical axes was created for visual interpretation [33].

### Stable isotope analysis

Frozen white muscle tissue samples were processed and analyzed for isotopic composition of C and N. Samples were left to lyophilize for 48 hours, or until it was clear the tissues were fully dried. Each dried sample was ground into a fine powder using a glass mortar and pestle and sent to the Stable Isotope Laboratory in Idaho State University’s (ISU) Department of Geosciences. Samples were analyzed at ISU using an ECS 4010 (Elemental Combustion System 4010) interfaced with a Delta V Advantage mass spectrometer through the COnFlo IV system at ISU. Four in-house standards (ISU Peptone, Costech Acetanilide, DORM-3, and ISU Glycine) were used to directly calibrate against international standards.

A weight ratio of C to N, which is commonly used as a proxy for lipid content, was calculated based on the percentages of C and N in each sample. To avoid biases to the δ^13^C (i.e. ^13^C:^12^C) that could be introduced if lipid content is too high, we omitted any tissue sample that showed greater than 5% lipid content, or a C:N weight ratio higher than 3.5 [34]. An ANOVA was used to determine the effects of location and year on δ^13^C and δ^15^N (i.e., ^15^N:^14^N), by comparing multiple means across different groups. Tukey’s Honest Significant Difference test was used as a post-hoc test to determine which locations and years were driving significant results.

Eltonian niche characteristics (ENCs), defined as those encompassing resource-consumer dynamics, can be used to evaluate the functional role of species or populations within a food web [35–37]. Six complementary ENCs were calculated for spatio-temporal groupings of *S*. *flavidus* to reflect the extent and direction of isotopic dispersion, which is ultimately driven by intraspecific variation [38, 39]. Metrics include carbon and nitrogen ranges (CR, NR), total convex hull area (TA), mean distance to the population centroid (CD), mean nearest neighbor distance (MNND), and standard deviation of MNND (SDMNND). CR and NR indicate the range of primary production sources, as well as the trophic diversity exhibited by different populations of *S*. *flavidus*. TA represents the total area of niche space occupied by the bivariate population means; thus, the combined variability of habitat (δ^13^C) and prey resources (δ^15^N) utilized by a *S*. *flavidus* population. CD is a measure of trophic diversity defined by the mean Euclidean distance between bivariate population means and the species centroid (bivariate mean for all populations). MNND defines the relative degree of individual trophic relatedness (i.e., packing, [38]) and is calculated as the mean Euclidean distance between each individual’s nearest neighbor. Finally, SDMNND accounts for sample size biases in MNND estimates and represents the relative degree of species evenness [38]. All statistical analyses were conducted in R (v. 3.3.2) using the laymanMetrics function in the SIBER package [39].

## Results

### Gut content analysis

**Fig 2:**
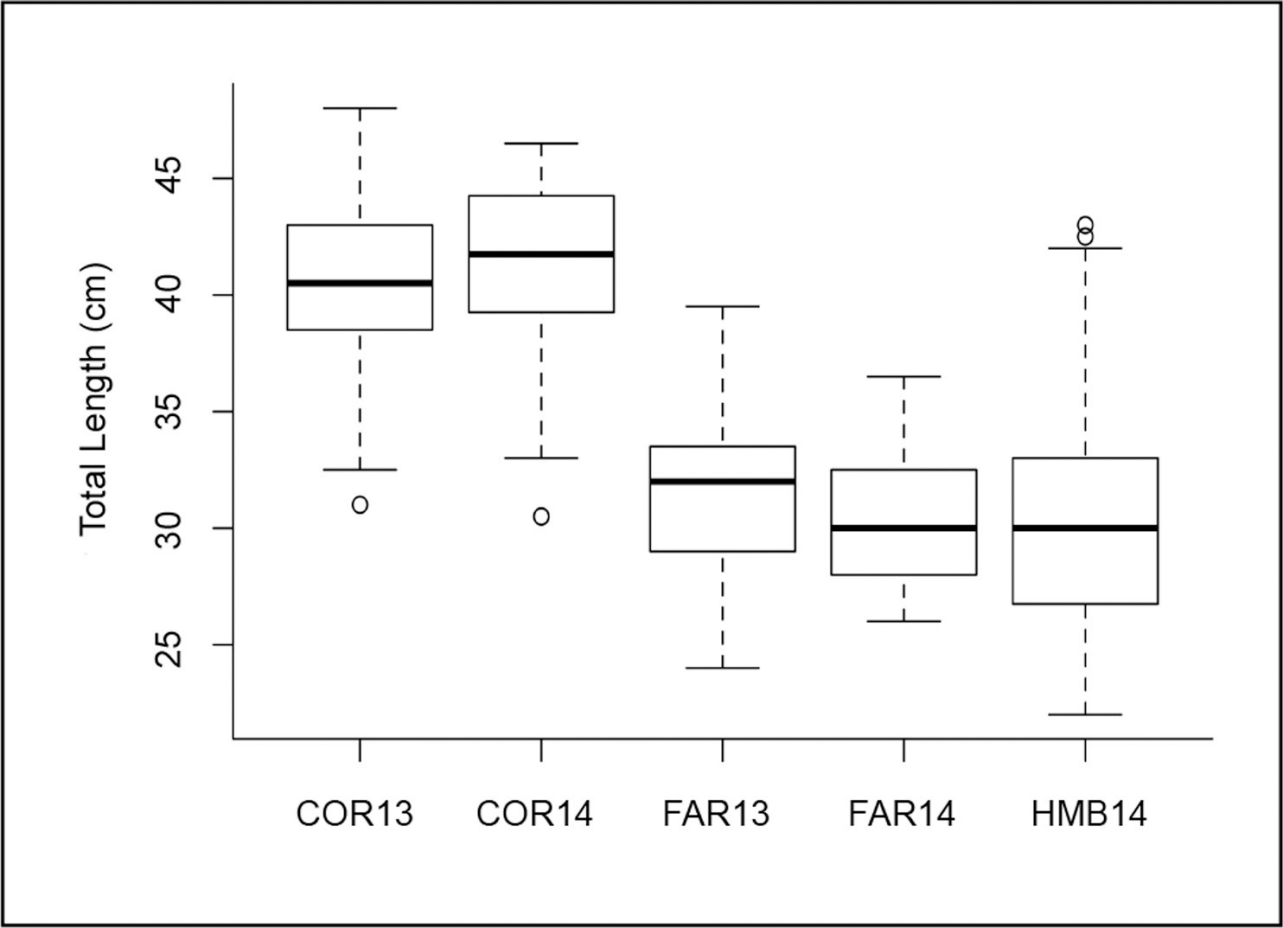
Box plot of total length (cm) of *Sebastes flavidus* caught by survey location and year. Bolded line inside each box represents the sample median. Horizontal lines at the top and bottom of each box are represented by +/- 1.5 * IQR (interquartile range). Whiskers represent the minimum and maximum extent of the data, excluding outliers. Open circles denote points that fall outside this range. COR = Cordell Bank, FAR = Farallon Islands, HMB = Half Moon Bay, 13 = 2013, 14 = 2014.

In total, 433 *Sebastes flavidus* ranging from 22–48 cm total length (mean ± sd = 34.5 ± 6.2 cm) were collected across two years and three locations (Table 1). Fish caught at Cordell Bank were larger (mean ± sd total length = 40.6 ± 3.8 cm in 2013, 40.7 ± 4.4 cm in 2014) than those caught at the Farallon Islands (mean ± sd total length = 31.4 ± 3.0 cm in 2013, 30.5 ± 2.5 cm in 2014) and Half Moon Bay (mean ± sd total length = 30.4 ± 4.7 cm in 2014) (Fig 2). An ANOVA indicated that there were significant differences in the lengths for each location and year (*F*_*3*,*250*_ = 112.0, *p* = <0.0001). *S*. *flavidus* caught at Cordell Bank in both years were larger than all other groups (Tukey’s Honest Significant Difference test, *p* < 0.001).

**Table 1:** Distribution of all collected yellowtail rockfish, *Sebastes flavidus*, the total number (n) of all stomach samples (total), the number with identifiable prey contents (Full), and the percentage of stomachs containing prey by location and year. Only stomachs with identifiable prey contents were used for analyses.

Location	Total (n)	Full (n)	Full (%)
**Cordell Bank 2013**	40	33	82.5
**Farallon Islands 2013**	56	41	73.2
**Cordell Bank 2014**	104	28	26.9
**Farallon Island 2014**	125	77	61.6
**Half Moon Bay 2014**	108	72	66.7
**Totals**	433	251	58.0

Of the 433 fish collected, 182 (42.0%) had empty stomachs, or stomachs containing only unknown solids and liquids; diets were analyzed for the remaining 251 fish. These 251 fish consisted of 97 males (38.7%), 105 females (41.8%), and 49 of indeterminate sex (19.5%). Based on generated cumulative prey curves for *S*. *flavidus*, the number of stomachs collected for dietary analysis was sufficient at the generalized level of prey categorization for each location and year (COR13: *b =* 0.033, FAR13: *b* = 0.049, COR14: *b* = 0.040, FAR14: *b* = 0.013, HMB14: *b* = 0.000, [40]). However, sample size was insufficient to adequately characterize *S*. *flavidus* diet at the lowest level of taxonomic identification for most spatio-temporal collections (*C13*: *b =* 0.124, F13: *b* = 0.098, C14: *b* = 0.151, F14: *b* = 0.014, H14: *b* = 0.057). Therefore, for all subsequent analyses, we utilized diet data grouped at the generalized level of prey categorization.

Among the 251 fish with prey in their stomachs, nearly 12,000 individual prey items were identified, comprising 18 taxonomic groups representing 4 phyla (Tables 2–4 and **S1 and S2 Datasets**). Utilizing the generalized prey categories to evaluate overall diet composition from a broad perspective, we determined that tunicates (39.7% PSIRI) were the most important prey items to the diet of *S*. *flavidus*, followed by crustaceans (18.4% PSIRI), and teleosts (13.4% PSIRI). Tunicates (45.8% FO), crustaceans (27.5% FO) and teleosts (17.5% FO) were eaten with the most regularity. Tunicates represented the highest prey-specific abundance by number (85.3% PN), whereas rockfish had the highest prey-specific abundance by weight (94.5% PW).

**Table 2:** **A) Diet composition of yellowtail rockfish, *Sebastes flavidus*, caught at Cordell Bank in 2013 (*n* = 33 stomachs). B) Diet composition of *S*. *flavidus* at Cordell Bank in 2014 (*n* = 28 stomachs)**.

**A) Lowest Taxonomic Level: Cordell 2013**				**%N**	**%PN**	**%W**	**%PW**	**%FO**	**%PSIRI**
**Chordata**									
	Actinopterygii								
		*Sebastes* spp.		3.0	100.0	3.0	100.0	3.0	3.0
			*Sebastes jordani*	8.2	44.8	15.5	85.1	18.2	11.8
		Unidentified fishes		14.1	51.5	23.8	87.1	27.3	18.9
	Tunicata								
		Salpidae spp.		29.1	87.3	20.5	61.4	33.3	24.8
**Arthropoda**									
	Crustacea								
		Euphausiacea		35.1	77.3	30.2	66.5	45.5	32.7
		Amphipoda		1.0	17.1	0.0	0.2	6.1	0.5
			Hyperiidea	1.0	33.3	0.0	0.3	3.0	0.5
		*Crangon* spp.		2.0	66.7	0.0	0.8	3.0	1.0
		Unidentified crustaceans		5.5	25.8	4.0	19.0	21.2	4.75
**Mollusca**									
	Cephalopoda								
		*Doryteuthis opalescens*		1.0	33.3	3.0	99.2	3.0	2.0
**B) Lowest Taxonomic Level: Cordell 2014**									
**Chordata**									
	Actinopterygii								
		*Citharichthys sordidus*		7.1	100.0	7.1	100.0	7.1	7.1
		Unidentified fishes		13.1	73.3	16.9	94.4	17.9	15.0
	Tunicata								
		Salpidae		9.3	65.0	9.6	67.3	14.3	9.5
		*Pyrosoma* spp.		0.1	14.3	35.5	14.3	100.0	14.3
		*Thetys vagina*		2.5	35.0	1.6	23.0	7.1	2.1
**Arthropoda**									
	Crustacea								
		Euphausiacea		15.3	85.8	11.0	61.8	17.9	13.2
		Amphipoda		14.3	66.7	13.6	63.6	21.4	14.0
			Hyperiidea	1.8	50.0	1.0	26.6	3.6	1.4
		*Crangon* spp.		3.6	100.0	3.6	100.0	3.6	3.6
		Unidentified crustaceans		0.7	20.0	0.0	0.4	3.6	0.4
**Mollusca**									
	Cephalopoda								
		*Doryteuthis opalescens*		14.4	80.9	17.7	99.0	17.9	16.1
**Annelida**									
	Polychaeta			3.6	100.0	3.6	100.0	3.6	3.6

**Table 3:** **A) Diet composition of yellowtail rockfish, *Sebastes flavidus*, caught at the Farallon Islands in 2013 (n = 41 stomachs). B) Diet composition of *S*. *flavidus* at the Farallon Islands in 2014 (n = 77 stomachs)**.

**A) Lowest Taxonomic Level: Farallons 2013**				**%N**	**%PN**	**%W**	**%PW**	**%FO**	**%PSIRI**
**Chordata**									
	Actinopterygii								
		*Sebastes* spp.							
			*Sebastes jordani*	0.0	0.5	1.9	77.8	2.4	1.0
	Tunicata								
		Salpidae		68.8	97.3	68.1	96.3	70.7	68.4
		*Pyrosoma* spp.		0.1	2.8	0.5	20.5	2.4	0.3
**Arthropoda**									
	Crustacea								
		Euphausiacea		15.3	78.4	14.7	75.1	19.5	15.0
		Amphipoda		0.3	1.1	0.1	0.3	24.4	0.2
			Hyperiidea	0.0	0.4	0.0	0.4	2.4	0.0
		*Crangon* spp.		0.1	0.9	0.1	0.3	14.6	0.1
		Unidentified crustaceans		13.0	88.9	12.3	84.0	14.6	12.7
**Mollusca**									
	Cephalopoda								
		*Doryteuthis opalescens*		2.4	100.0	2.4	100.0	2.4	2.4
**B) Lowest Taxonomic Level: Farallons 2014**									
**Chordata**									
	Actinopterygii								
		*Sebastes* spp.		4.1	79.2	5.1	97.7	5.2	4.6
			*Sebastes jordani*	13.0	100.0	13.0	100.0	13.0	13.0
		Unidentified fishes		4.0	77.5	4.2	81.1	5.2	4.1
	Tunicata								
		Salpidae		17.7	64.8	23.6	86.5	27.3	20.6
		*Pyrosoma* spp.		0.0	0.5	10.2	2.7	51.9	5.2
**Arthropoda**									
	Crustacea								
		Euphausiacea		5.2	80.0	4.4	68.1	6.5	4.8
		Amphipoda		3.2	41.0	3.5	44.6	7.8	3.3
			Hyperiidea	1.6	40.4	1.2	31.6	3.9	1.4
			*Vibilia* spp.	1.3	17.0	0.8	10.5	7.8	1.1
		*Crangon* spp.		2.0	75.0	1.4	53.9	2.6	1.7
		Unidentified crustaceans		46.2	82.6	38.8	69.5	55.8	42.5
	Cephalopoda								
		*Doryteuthis opalescens*		1.3	100	1.3	100	1.3	1.3

**Table 4:** Diet composition of yellowtail rockfish, *Sebastes flavidus*, caught at Half Moon Bay in 2014 (*n* = 72 stomachs).

Lowest Taxonomic Level: Half Moon Bay 2014				%N	%PN	%W	%PW	%FO	%PSIRI
**Chordata**									
	Actinopterygii								
		Unidentified fishes		24.6	73.9	25.5	76.6	33.3	25.1
	Tunicata								
		Salpidae		40.7	86.2	35.7	75.5	47.2	38.2
		*Pyrosoma* spp.		13.4	42.0	18.0	56.4	31.9	15.7
**Arthropoda**									
	Crustacea								
		Isopoda		1.4	100.0	1.4	100.0	1.4	1.4
		Euphausiacea		5.8	52.3	4.3	38.8	11.1	5.1
		Amphipoda		0.0	1.5	0.0	0.2	1.4	0.0
		Caprellidae		0.1	10.0	0.0	2.4	1.4	0.1
		Hyperiidea							
			*Vibilia* spp.	0.7	24.4	0.2	5.4	2.8	0.4
		*Crangon* spp.		1.4	100.0	1.4	100.0	1.4	1.4
		Unidentified crustaceans		5.0	51.0	3.0	31.2	9.7	4.0
**Mollusca**									
	Cephalopoda								
		*Doryteuthis opalescens*		4.2	50.3	7.3	87.4	8.3	5.7
	Gastropoda								
		*Pterotracheoida* spp.		0.5	17.2	0.0	0.4	2.8	0.2
**Annelida**									
	Polychaeta			2.2	39.4	3.2	57.4	5.6	2.7

**Fig 3:**
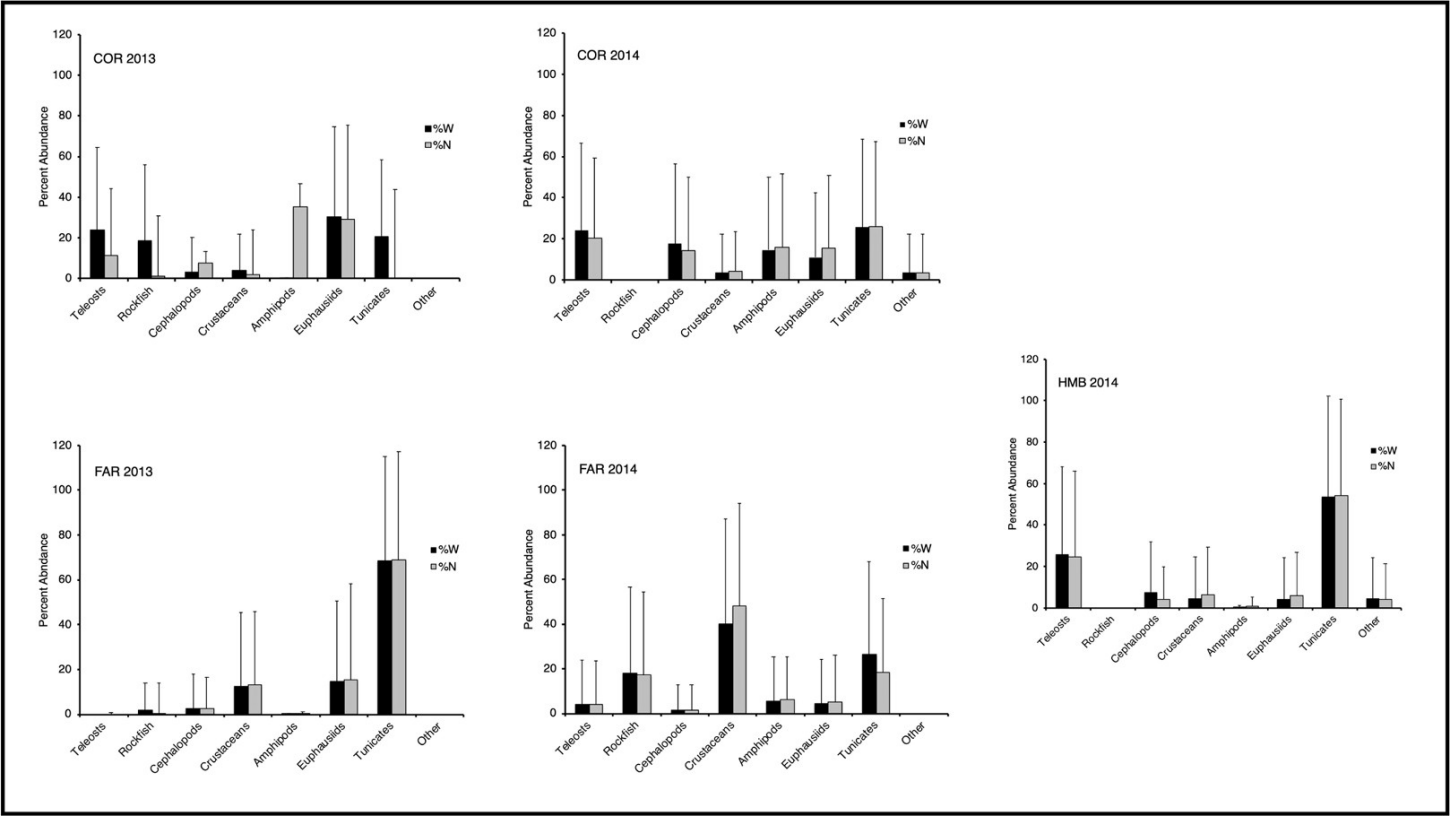
Graphs of percent weight (%W) and number (%N) of the 8 major prey categories in the diet of *Sebastes flavidus* caught off central California in 2013 and 2014. Error bars represent standard deviations of the mean (sample size = 251 fish). See Tables 2–4 for prey categories. Abbreviations: COR = Cordell Bank, FAR = Farallon Islands, HMB = Half Moon Bay.

Euphausiids, tunicates, and fishes dominated diets of *S*. *flavidus* at Cordell Bank in 2013, whereas euphausiids were relatively less important than those and other prey groups (squids, amphipods) in 2014 (Table 2A and 2B and Fig 3). Within the tunicate group, only salps were consumed at Cordell Bank during 2013 (24.8% PSIRI). Pyrosomes (14.3% PSIRI) were incorporated into the diet in 2014 and were relatively more important than salps in that year (11.6% PSIRI). Additionally, rockfishes were not recorded in stomach contents from 2014 and polychaetes were not ingested during 2013. The most important prey groups found in stomachs from 2014 were those most frequently consumed during 2013 (Table 2A and 2B). All *S*. *flavidus* sampled at Cordell Bank in 2014 preyed on pyrosomes, whereas amphipods, the next most frequently consumed prey taxon, were only present in 21.4% of samples. During 2013, relatively large prey items (i.e., unidentified fishes, *Doryteuthis opalescens*) exhibited greater %W values and much greater %PW values; conversely, %N and especially %PN values were elevated for smaller prey types, such as crustaceans. Far fewer prey items were identified during 2014 (n = 86) than during 2013 (n = 1584); correspondingly, %PN and %PW values were inflated and more similar for most taxa during 2014 (Table 2A and 2B).

At the Farallon Islands, the diet of *S*. *flavidus* varied considerably between 2013 and 2014 (Fig 3). Tunicates (68.7% PSIRI), comprising almost exclusively salps, were the most important prey group during 2013, followed by euphausiids (15.0% PSIRI) and unidentified crustaceans (12.7% PSIRI) (Table 3A). The relative importance of tunicates (25.8% PSIRI) and euphausiids (4.8% PSIRI) declined in 2014, whereas the relative importance of unidentified crustaceans (42.5% PSIRI), rockfishes (17.6% PSIRI), amphipods (5.8% PSIRI), and pyrosomes (5.2% PSIRI) increased substantially (Table 3A and 3B). %FO was generally similar to %PSIRI for most taxa in both years; however, %FO was an order of magnitude greater than %PSIRI for pyrosomes during 2014 and two orders of magnitude greater than %PSIRI for amphipods and *Crangon* spp. during 2013 (Table 3A and 3B). Numerical (%N, %PN) and gravimetric (%W, %PW) metrics generally were similar within taxa, with the exception of rockfishes and pyrosomes, which had substantially greater gravimetric representation during both years, and most crustacean taxa, which had slightly greater numerical representation during 2014 (Table 3A and 3B). As documented at Cordell Bank, substantially fewer prey items were identified from 2014 samples (*n* = 561) as compared to 2013 (*n* = 8,580).

Tunicates dominated stomach contents of fish caught in Half Moon Bay during 2014 by all non-prey specific metrics (Table 4 and Fig 3). Within the tunicates, salps (38.2% PSIRI) were more important prey than pyrosomes (15.7% PSIRI). Teleosts also were a substantial component of diet at this site, ranking second to tunicates for all non-prey specific metrics. Squid (*D*. *opalescens*), euphausiids, and unidentified crustaceans were supplemental prey taxa of similar dietary importance (4.0–5.7% PSIRI). Furthermore, these prey groups were consumed by a similar proportion of the population (8.3–11.1% FO) (Table 4). *D*. *opalescens*, *Pterotracheoida* spp., and *Pyrosoma* spp. contributed greater gravimetric (%W, %PW) dietary contributions, whereas most crustaceans contributed greater numerical (%N, %PN) dietary contributions. More prey items were identified at Half Moon Bay during 2014 (n = 1,120) as compared to either of the other collection sites.

Mean trophic level estimates of *S*. *flavidus* diets among all years and locations ranged from 3.25 to 4.24, with an overall mean of 3.69 ± 0.36 sd. The minimum (3.25) and maximum (4.24) values of this range corresponded to euphausiid-exclusive or fish-exclusive diets, respectively. The mean trophic level at Cordell Bank was 3.75 ± 0.44 sd in 2013 and 3.88 ± 0.39 sd in 2014. The mean trophic level at the Farallon Islands was 3.48 ± 0.18 sd in 2013 and 3.66 ± 0.35 sd in 2014, but the interquartile range was much greater in 2014, indicating that *S*. *flavidus* was eating more fish in 2014. The mean trophic level of the Half Moon Bay population in 2014 was estimated at 3.74 ± 0.35 sd. Mean trophic level ranks differed significantly among years and locations (*X*^*2*^ = 18.46, *df* = 4, *p* = 0.001). Specifically, mean ranks from all other regions were significantly greater than those of the Farallon Islands population in 2013 (*P* < 0.05) but not significantly different from each other (*P* ≥ 0.05) as a result of substantial trophic level variability within and among populations. There was no significant relationship between trophic level and total length by sex using pooled data from all regions (ANCOVA, *F*_*1*, *191*_ = 0.172, *p* = 0.679).

Results of the PERMANOVA analysis indicated considerable dietary variability among and within explanatory variables. The variables incorporated in the best-fit %N and %W PERMANOVA models for *S*. *flavidus* off central California included Location, Longitude, Year, Month, and Depth (Table 5). Although all interaction terms were significant when assessed independently, they did not contribute to the final %N or %W models. Within the %N model, Location was of greatest relative importance in explaining dietary differences, followed by Longitude, Year, Month, and Depth, respectively (Table 5). In combination, these variables explained 20% of dietary variation in the %N data set. In the best-fit model for %W, Month and Location variables contributed most towards explaining dietary differences, followed by Longitude, Year, and Depth variables, respectively. This combination of variables explained 24% of the overall dietary variability in the %W data set. Permutation tests for homogeneity of multivariate dispersions showed that Location (*P* = 0.001) and Year (*P* = 0.009) categories differed significantly for %N, and Location (*P* = 0.001) and Depth (*P* = 0.001) were significantly different for %W (Table 5).

**Table 5:** Final PERMANOVA models of diet composition among several response variables for the yellowtail rockfish, *Sebastes flavidus*, at Cordell Bank, the Farallon Islands, and Half Moon Bay in 2013 and 2014 (n = 251 stomachs).

		%N			%W		
Variables	*df*	*F*	*r*^*2*^	*P*	*F*	*r*^*2*^	*P*
**Location**	2	15.44	0.103	**0.001**	12.16	0.077	**0.001**
**Longitude**	1	9.01	0.030	0.001	11.26	0.036	0.001
**Year**	1	7.77	0.026	**0.001**	9.17	0.029	0.001
**Month**	2	4.92	0.032	0.001	13.77	0.087	0.001
**Depth**	1	4.16	0.013	0.006	4.56	0.014	**0.003**
**Residuals**	246		0.794			0.757	

Variables are arranged based on the combined value of their F-statistics to indicate the relative magnitude of significant differences. Degrees of freedom (*df*), F-statistic, amount of variability explained (*r*^*2*^), and *P* value are included for percent number (%N) and percent weight (%W) data. *P* values that are in bold indicate significant results from multivariate homogeneity of group dispersion tests to the *P* < 0.01 level.

**Fig 4:**
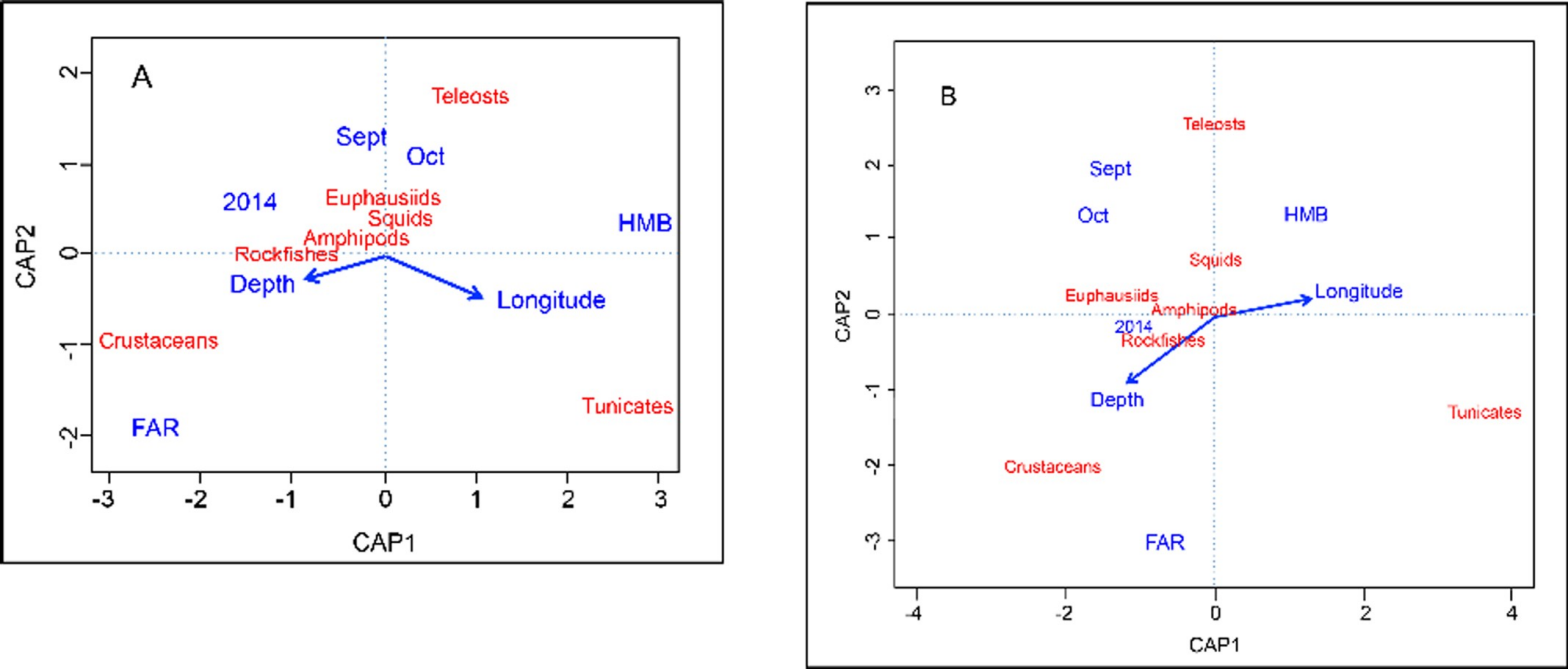
**Biplot of first two Canonical analysis of principal component (CAP) axes for (A) %N and (B) %W models.** Generalized *Sebastes flavidus* prey categories are shown in red and significant explanatory variables and variable categories are shown in blue. Vectors indicate increasing gradients for continuous variables. HMB = Half Moon Bay sampling site and FAR = Farallon Islands sampling site.

CAP ordinations yielded results that were largely consistent with PERMANOVA models and provided interpretive support by associating prey taxa with specific spatial and temporal variables. The %N model was significant (*F* = 8.41, *P* = 0.001) and explained 21.5% of the variance in the data set, with the first two axes accounting for 16.7% of the total (CAP1: *F* = 31.94, *P* = 0.001, 11.2%, CAP2: *F* = 15.93, *P* = 0.001, 5.6%). All input variables except Length were significant (*P* < 0.01). The first canonical axis indicated that the numerical proportions of Crustaceans and Tunicates in the diet were negatively correlated (Fig 4A). High relative numerical proportions of Crustaceans and Teleosts were observed in *S*. *flavidus* diets at the Farallon Islands and at deeper depths and proportionally greater amounts of Tunicates and Rockfishes were consumed by fish collected at Half Moon Bay and with increasing longitude. CAP2 primarily indicated greater relative proportions of Teleosts in fish diets during September and October, and greater consumption of tunicates and crustaceans at the Farallon Islands (Fig 4A). The %W CAP ordination was significant (*F* = 9.83, *P* = 0.001) and similar to the %N ordination. The %W model explained 24.4% of the variance in the data set, with CAP1 (*F* = 39.87, *P* = 0.001, 13.3%) and CAP2 (*F* = 21.76, *P* = 0.001, 7.1%) accounting for 20.4% of the total. All input variables except Length were significant (*P* < 0.01). CAP1 indicated a greater reliance on tunicates at Half Moon Bay and with increasing longitude, and larger gravimetric proportions of Crustaceans, Euphausiids, and Rockfishes that were less strongly associated with the remaining spatio-temporal variables (Fig 4B). The second canonical axis indicated that fishes collected during September and October and those from Half Moon Bay consumed a greater gravimetric proportion of Teleosts and a lower proportion of Crustaceans and Tunicates than those collected from the Farallon Islands and at deeper depths (Fig 4B).

### Stable isotope analysis

**Fig 5:**
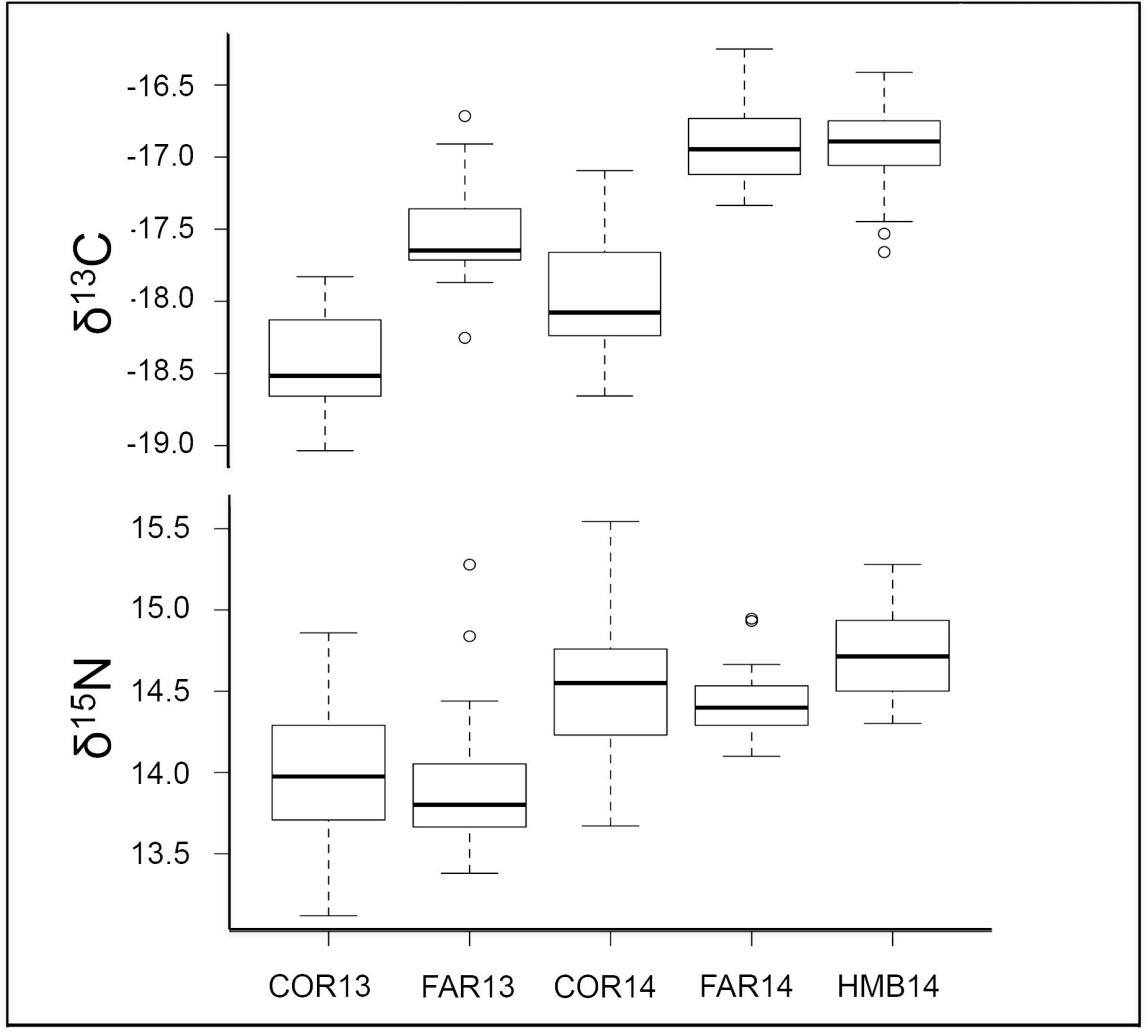
Box plot of the δ^13^C and δ^15^N distributions of *Sebastes flavidus* white muscle tissue for all locations and years. Bolded line inside each box represents the sample median. Horizontal lines at the top and bottom of each box are represented by +/- 1.5 * IQR (interquartile range). Whiskers represent the minimum and maximum extent of the data, excluding outliers. Open circles denote points that fall outside this range. COR = Cordell Bank, FAR = Farallon Islands, HMB = Half Moon Bay, 13 = 2013, 14 = 2014.

Substantial spatial, temporal, and individual variability was evident in stable isotope signatures. Overall, mean δ^13^C in samples ranged between -19.0‰ and -16.3‰, whereas δ^15^N values ranged from 13.1–15.5‰ (**S3 Dataset**). The range of C:N ratios was 3.1–3.8. Significant spatio-temporal differences were evident in both δ^13^C (*F*_*4*,*145*_ = 30.91, *p* < 0.001, Fig 5A) and δ^15^N (*F*_*4*,*145*_ = 115.7, *p* < 0.001, Fig 5B); however, δ^13^C and δ^15^N signatures were each more negative in 2013 than in 2014 at the same locations (Fig 5).

ENCs indicated spatio-temporal variability in feeding behavior among central California *S*. *flavidus* populations. Carbon ranges decreased on a gradient from offshore (Cordell Bank) to inshore (Half Moon Bay), whereas nitrogen ranges increased substantially at Cordell Bank and Farallon Islands between 2013 and 2014 (Table 6). Niche space (TA) varied by location and year with no clear trend. Trophic diversity (CD) declined from 2013 to 2014 at sites that were sampled in both years (Cordell Bank, Half Moon Bay). MNND values varied among locations and years and did not suggest an obvious trend; however, SDMNND indicated that individual variability was substantial for MNND.

**Table 6:** Eltonian Niche Characteristics (ENCs) calculated from δ^13^C and δ^15^N data for populations of yellowtail rockfish, *Sebastes flavidus*, sampled at three locations off central California in 2013 and 2014.

Location and Year	n	CR	NR	TA	CD	MNND	SDNND
**Cordell Bank 2013**	30	1.74	1.26	1.07	0.50	0.13	0.09
**Farallon Islands 2013**	30	1.08	0.85	1.46	0.44	0.17	0.15
**Cordell Bank 2014**	28	1.57	1.87	1.22	0.45	0.15	0.13
**Farallon Islands 2014**	30	1.53	1.90	0.69	0.31	0.10	0.09
**Half Moon Bay 2014**	28	1.25	0.98	0.96	0.38	0.12	0.08

n = sample size, CR = carbon range, NR = nitrogen range, TA = total convex hull area, CD = mean distance to the population centroid, MNND = mean nearest neighbor distance, SDMNND = standard deviation of MNND.

## Discussion

Our results provide new insights into the trophic ecology of the yellowtail rockfish, *Sebastes flavidus*, a common and widespread mesopredator in the California Current Large Marine Ecosystem. Gelatinous zooplankton (i.e., tunicates) were of greater dietary importance to the populations of *S*. *flavidus* in our study than previously noted [10, 12, 13, 41], and the incorporation of pyrosomes into the prey spectrum was a novel finding. Since 2013, and likely initiated by rising sea surface temperatures and northward flow of subtropical waters, the abundance of the most common tunicates captured in pelagic trawl surveys in the California Current (i.e., *Thetys vagina*, *Pyrosoma atlanticum*) has increased markedly [42]. Furthermore, a shift in the relative contribution of salps and pyrosomes to *S*. *flavidus* diets between 2013 (salps) and 2014 (pyrosomes) coincides with a shift in the relative abundance of these species off central California [42]. A substantial reduction in euphausiid consumption and greater piscivory by sampled *S*. *flavidus* populations during 2014, including elevated trophic positions by stomach content and stable isotope analysis, similarly appears to be driven by a concurrent coastwide decrease in euphausiid abundance [3, 7]. The spatial and temporal dietary variability evident in our stomach content and stable isotope results suggests that 1) spatial dietary differences (i.e., by location, longitude, or distance offshore) are pronounced among *S*. *flavidus* populations, and 2) prey switching in *S*. *flavidus* among all sites between 2013 and 2014 was driven by changes in the species composition of the pelagic forage base at the onset of the recent marine heat wave.

Offshore sea surface temperatures (SSTs) in the NE Pacific were 2.5°C higher than normal in the winter of 2013–2014, with the largest anomalies exceeding three standard deviations [43]. The warm anomalies in California began after we finished sampling in October 2013, and by May 2014 the region of anomalously warm SST covered California shelf waters. The Pacific Decadal Oscillation index (PDO, [44]) rose throughout 2014, indicative of an increase in warm coastal waters [45]. Fall 2014 SSTs in central California were some of the highest recorded since 1990 [45]. The warm SSTs were associated with weaker than normal winds and reduced coastal upwelling. Monthly means of the daily upwelling index indicated strong upwelling for the entire year of 2013, but the cumulative upwelling index was lower than the long-term mean in the second half of 2014 [45].

Reduced upwelling and higher SSTs during the 2014–2016 marine heat wave caused a dramatic change in species composition and abundance of many zooplankton and marine invertebrate populations in the California Current Ecosystem. The productivity of the system, based on the standing stock of chlorophyll a, a proxy for system productivity, was much lower in 2014 compared to 2013 [45]. Between the fall of 2013 and 2014, the arrival of warm waters caused a species shift from subarctic copepods, which contain large quantities of fatty acids, to smaller, less nutritious subtropical copepods [1, 46]. Subarctic euphausiid populations also were negatively affected by the warm water conditions. During the marine heat wave, the coastwide abundances of large *Euphausia pacifica* were lower than in any of the past 18 years [47, 48], and a similar pattern was suggested for *Thysanoessa spinifera* [48]. Furthermore, Sakuma et al. [2] described anomalously high abundances of gelatinous zooplankton from a midwater micronekton-trawl survey in the spring of 2015 in central California. They reported that abundances of salps and pyrosomes were comparable to the long-term averages in 2013, but by 2015 they recorded the highest catches of pyrosomes ever observed in their surveys. Conversely, the abundances of krill, especially *T*. *spinifera*, the most common nearshore euphausiid in our study region, were at or below long-term averages.

Fishes in the California Current exhibit substantial dietary shifts between cold periods and warm periods. For instance, Brodeur et al. [49] compared the diets of small pelagic fishes caught in trawl surveys in the anomalously warm springs of 2015 and 2016 off the Oregon and Washington coasts with the diets of those species caught in normal and anomalously cool periods and found marked dietary variability. They surveyed some of the most commonly occurring pelagic fishes off California, such as *Engraulis mordax* (Northern anchovy), *Sardinops sagax* (Pacific sardine), and *Trachurus symmetricus* (Jack mackerel). Small planktonic crustaceans were the main prey items in those fishes for most years examined; however, gelatinous zooplankton were consumed in much higher quantities in warm water years. They suggested that in periods of warm water, fishes may feed on gelatinous zooplankton in the absence of more preferred prey resources.

Gelatinous zooplankton are known to be a prey source for many fish species in the northeast Pacific [41], but the overall importance in food webs is often underrepresented. One reason for this is because gelatinous zooplankton abundance may not be adequately represented in stomach samples due to a more rapid digestion process or sampling methods. Previous studies have shown that *S*. *flavidus* tend to consume larger abundances of gelatinous zooplankton when available in their habitat [12, 41]. Knowing the role that environmental factors and changing ocean conditions play on the availability of this prey category influences our understanding of predator feeding strategy and energy transfer throughout the food web.

Three different feeding strategies have been described for post-recruitment juvenile and adult *S*. *flavidus*, depending on when and where the study was conducted. In a study of diets of juvenile and adult *S*. *flavidus* in the eastern Gulf of Alaska, Rosenthal et al. [11] concluded that this species was a dietary specialist because it fed most heavily on fishes. This supposition supported limited earlier work by Pereyra [50], who reported that the northern lampfish, *Stenobrachius leucopsarus*, dominated the diets of 22 adult fish collected at the head of Astoria Canyon, Oregon. Also in Oregon, however, Brodeur and Pearcy [10] suggested that late juvenile and adult *S*. *flavidus* from southern Oregon, USA to Vancouver, British Columbia, Canada were generalists. Although euphausiids, principally *E*. *pacifica* and *Thysanoessa spinifera*, were the principal prey group recorded by all metrics in their study, fishes and a wide suite of supplemental prey taxa also contributed substantially to a spatial and temporally dynamic diet composition. Furthermore, *S*. *flavidus* had the greatest overall niche breadth and number of prey taxa recorded in comparisons among five rockfishes [10]. Similarly, other researchers [51] have reported generalist feeding habits for *S*. *flavidus* from Puget Sound, but smaller juveniles primarily consumed copepods, mysids, fishes, crab larvae, and chaetognaths.

Late stage juvenile and adult *S*. *flavidus* were collected by Lee and Sampson [12] in the same general locations as the study by Brodeur and Pearcy [10], and they reported that, by weight, euphausiids (20%) were much less important than fishes (41%), presumably as a result of the lower abundance of euphausiids during El Niño conditions. Also, they reported that a variety of gelatinous zooplankton species comprised 22% of *S*. *flavidus* diet by weight in 1998 [8]. They indicated that patterns in the diet of *S*. *flavidus* were associated with geographical and temporal factors, and suggested that *S*. *flavidus* should be considered an opportunistic feeder. This conclusion is consistent with the prior findings of Whipple [13], who indicated seasonal shifts in diets of *S*. *flavidus* at Heceta Bank, Oregon from salps in winter to euphausiids during spring and summer upwelling periods. Additionally, Lorz et al. [52] indicated opportunistic incorporation of benthic prey and differential consumption of fishes and crustaceans between late juveniles and adults sampled at Queen Charlotte Sound, Canada and off the coast of Washington, USA.

In 1994, Gerking [53] suggested that a fish species might switch from specialist to generalist during a period when food abundance declines abruptly or competition increases. Both the El Niño event that occurred during the study by Lee and Sampson [12] and the marine heat wave that occurred in the California Current Ecosystem from 2014–2016 caused precipitous declines in euphausiid abundances in the California Current Ecosystem. Based on the decline in zooplankton abundances in central California in 2014, we hypothesized that if *S*. *flavidus* are stenophagous as Rosenthal et al. [11] suggested, then their diets in 2014 would contain a higher proportion of fishes than we observed in 2013. Our rationale was that, in the absence of easily caught, high-lipid content euphausiids, *S*. *flavidus* would consume fishes that are of higher caloric value than gelatinous zooplankton. Alternatively, if *S*. *flavidus* are euryphagous as Brodeur and Pearcy [10] suggested, then we would expect *S*. *flavidus* diets to greatly differ between 2013 and 2014, based on the large changes occurring due to the marine heat wave.

During the marine heat wave, gelatinous zooplankton were more abundant [1, 2] and consumed in much higher quantities by small pelagic fishes than in previous colder years [49]. Although temporal prey switching in our sampled populations appears to be evident in association with the marine heat wave, the main sources of dietary variability were spatial factors (location and longitude). Furthermore, individual dietary variability was substantial at all locations, as evidenced by the amount of dispersion around our mean diet composition estimates and our δ^13^C and δ^13^N signatures. These findings suggest considerable dietary plasticity in *S*. *flavidus* and support the notion that it is a eurypahgous predator. More research is necessary to determine if the prey spectrum consumed during 2013 represents a baseline condition and to robustly evaluate the degree of environmental coupling evident in the diet of the Yellowtail Rockfish.

The dietary importance of euphausiids in *S*. *flavidus* diet declined between 2013 and 2014, further supporting the strong coupling of this dietary variability with environmental dynamism. We sampled before and at the beginning of the marine heat wave, but other studies have shown this trend continuing in 2015 and 2016 where gelatinous zooplankton availability remained high, and euphausiid availability was low compared to previously sampled years [2, 3]. Thus, we expect that the diets of *S*. *flavidus* were probably even more anomalously shifted towards gelatinous zooplankton during the height of the marine heat wave.

Our combined stomach content and stable isotope results further support a mesopredatory role for Yellowtail Rockfish. Among *Sebastes* congeners sampled in association with Astoria Canyon, Oregon, δ^15^N and δ^13^C values were enriched for largely piscivorous *S*. *pauscispinis* compared to more omnivorous *S*. *flavidus* and *S*. *entomelas* [54]. The slightly greater δ^15^N determined for the Astoria Canyon *S*. *flavidus* population probably reflects an elevated trophic level in association with the larger fish sampled in that study as compared to ours [54]. Omnviory, as demonstrated for *S*. *flavidus* in this study and others, is especially important in the California Current, where most predatory nekton species consist of mesopredators that complement diets of fishes and squids with typically large and abundant zooplankton (especially euphausiids) [55]. Additionally, the δ^13^C values we observed among central California *S*. *flavidus* populations (δ^13^C HMB > δ^13^C FAR > δ^13^C COR) are consistent with the findings of Miller et al. [56], who demonstrated increasing δ^13^C enrichment with distance offshore in the northern California Current. We therefore suggest that the δ^13^C values we observed at replicated sampling sites during 2014 probably reflected elevated predation on a more oceanic prey base.

The presumption that *S*. *flavidus* feeds opportunistically best explains the dietary variability that we observed in Central California populations of *S*. *flavidus*. Given the known shifts in forage species (e.g., euphausiids) during the course of our study [42, 47] and the relatively low caloric value of tunicates when compared to euphausiids [57, 58], our results suggest that, in accordance with optimal foraging theory, it was more energetically costly to undertake foraging excursions for fishes than to consume high local densities of tunicates. Therefore, opportunistic prey switching in *S*. *flavidus* appears to have been driven by local densities of forage species, their ease of capture and ingestion, and their relative energetic importance. This idea is supported by our multivariate and stable isotope results, which indicate dietary differences among *S*. *flavidus* populations at relatively small spatial and temporal scales.

A synthesis of our results and prior research on *S*. *flavidus* enables a comprehensive description of the ecology and functional predatory role of this species. *S*. *flavidus* occurs in schools throughout the water column and to depths of 549 m, but typically occupies midwater regions (90–180 m) over high relief, mid and outer continental shelf seafloors [8]. Outer shelf populations commonly feed on midwater organisms (e.g., euphausiids and myctophids) that are advected shoreward during vertical migrations [50, 52]. Increased euphausiid consumption at our outer continental shelf sites (Cordell Bank, Farallon Islands) is suggested but the absence of 2013 data for Half Moon Bay and the onset of the marine heat wave preclude a definitive conclusion.

*S*. *flavidus* is one of the most mobile of all rockfishes. It is capable of making long distance movements [59, 60], rapid vertical movements without suffering barotrauma, and has a strong homing tendency [8, 61]. This motility probably allows *S*. *flavidus* to find and exploit dynamic forage hot spots that are driven by oceanographic conditions to create a heterogeneous zooplankton preyscape on a variety of spatial scales [62, 63]. Furthermore, this spatial heterogeneity in forage density is probably the source of the spatial and temporal dietary differences we observed in this study.

Our results and prior research suggest coupling between the availability of pelagic forage items and their utilization by *S*. *flavidus*. As an opportunistic predator with a broad prey spectrum, *S*. *flavidus* diet composition appears to represent the available preyscape at the time and location of specimen collection [64]. Consequently, monitoring *S*. *flavidus* diet can indirectly capture the spatio-temporal dynamism in the relative abundance of pelagic forage species. *S*. *flavidus* therefore may be an important ecosystem indicator species in the California Current.

## Conclusions

Yellowtail rockfish, *Sebastes flavidus* caught in central California between 2013 and 2014 were opportunistic feeders that consumed a wide variety of transitory pelagic prey items. The stomach contents of *S*. *flavidus* caught in our study greatly varied by location and by sampling date. The marine heat wave that occurred in the eastern Pacific Ocean for two years after 2013 contributed to this variability because of dramatic changes in the composition and abundance of prey items typically eaten by *S*. *flavidus*. This species, and presumably other long-lived rockfishes, was able to adapt to feed on the prey items that were most abundant during the heat wave even though the abundant food items were not as energetically rich as their normal prey items. Both stomach content analysis and stable isotope analysis provided evidence that *S*. *flavidus* diets are inherently variable and the species displays foraging plasticity with respect to time and space.

## Supporting information

S1 Dataset Numbers of prey items of each generalized prey category found in individual *Sebastes flavidus* stomachs. (CSV)

S2 Dataset Weights of prey items (g) of each generalized prey category found in individual *Sebastes flavidus* stomachs. (CSV)

S3 Dataset The δ15N and δ13C values (‰) of muscle tissue collected from captured *Sebastes flavidus*. (CSV)
